# Study on Preparation and Performance of Acid pH-Responsive Intelligent Self-Healing Coating

**DOI:** 10.3390/polym16172473

**Published:** 2024-08-30

**Authors:** Jianguo Liu, Feiyu Chen, Qiaosheng Zhang, Xiao Xing, Gan Cui

**Affiliations:** 1 College of Pipeline and Civil Engineering China University of Petroleum (East China), No. 66, West Changjiang Road, Huangdao District, Qingdao 266580, China; liujianguo@upc.edu.cn (J.L.); mitsuheshikiri@gmail.com (F.C.); 20170079@upc.edu.cn (X.X.); 2 Changqing Engineering Design Co., Ltd., Xi’an 710000, China; zqs3_cq@petrochina.com.cn

**Keywords:** pH response, intelligent self-healing, microcapsules, chitosan

## Abstract

In this paper, microcapsules with acidic pH stimulus responsiveness were prepared through a one-step in situ polymerization method and a layer-by-layer assembly method. The effects of factors such as chitosan (CS) concentration, polymerization time, polymerization process temperature, and the number of polymerization layers on the performance of microcapsules were explored, and microcapsules with optimal performance were prepared and added to the epoxy coating. The morphology and structure of the microcapsules were characterized by scanning electron microscopy, Fourier transform infrared spectroscopy, and zeta potential testing. The thermal stability and sustained release properties of the microcapsules were studied through thermogravimetric analysis and sustained release curve testing. Through scratch experiments, immersion experiments, salt spray experiments, and electrochemical impedance spectroscopy tests, the impact of the added amount of microcapsules on the self-healing performance and anti-corrosion performance of the coating in complex environments was explored. The results show that the optimal preparation process of acidic pH-responsive microcapsules requires that the concentration of chitosan is 2 mg/mL, the polymerization time of the polyelectrolyte layer is 8 h, the heating temperature during the polymerization process is 75 °C, and the number of polyelectrolyte layers is three. The prepared acidic pH-responsive microcapsules have good morphology, pH sensitivity, and thermal stability. The average particle size is approximately 203 μm, the drug loading rate reaches 59.74%, and the encapsulation rate reaches 63.99%. The optimal added amount of the acidic pH-responsive microcapsule coating is 15 wt%. The coating has a dual-trigger mechanism underlying it stimulus response capability and has an obvious stimulus response to acidic pH. It can inhibit corrosion in non-scratch areas, and its anti-corrosion ability is significantly stronger than that of epoxy coatings and ordinary self-healing coatings. The coating has a stronger repair effect and anti-corrosion ability when the environmental pH becomes acidic.

## 1. Introduction

Applying paint to metal surfaces is a common means of preventing corrosion. Commonly used coatings such as epoxy resin and polyester have good anti-corrosion properties and can effectively extend the life of materials [1]. Microcracks are the main cause of coating failure. Microcapsule self-healing coatings can achieve self-healing at cracks, but most common microcapsule self-healing coatings only respond to external mechanical stimulation. To solve this problem, scholars have proposed self-healing coatings with new stimulus response mechanisms. According to different stimulus response mechanisms, stimulus-responsive self-healing coatings can be divided into redox response [2], light response [3,4,5,6,7], pH response [8,9], temperature response [10,11,12,13], ionic strength response [14,15], etc.

The self-healing coating, which intelligently releases loaded corrosion inhibitors in response to pH signal changes, is a promising anti-corrosive coating with sufficient corrosion resistance and has great application prospects in the field of anti-corrosion. Currently, polyelectrolyte assembly technology [16,17,18], supramolecular chemistry [19], electrospinning, end blocking [20,21], and surface encapsulation technology [22] have been used to construct pH-responsive shells. Polyelectrolyte assembly technology is one of the most commonly used methods for constructing pH-responsive materials. As a polymer carrying positively charged or negatively ionizing groups [23], the polyelectrolyte dissociates in polar solvents such as water, leaving a charge on the polymer chain and releasing an anti-charge in the solution. Charged groups on polyelectrolytes can provide a sensing mechanism for changing physical or chemical properties around them. Polyelectrolytes can adapt to their surroundings, regulate the transport of ions and molecules, and change the response of different substances to external stimuli. Using the electrostatic interaction between charged groups on the polyelectrolyte, the polyelectrolyte with opposite charge can be easily assembled using the layer-by-layer assembly method; the multilayer polyelectrolyte membranes assembled layer by layer can respond well to ambient pH stimulation to restore the anti-corrosive properties and integrity of the coating [24,25]. At the same time, At the same time, polyelectrolyte membrane can interact with various organic or inorganic molecules through electrostatic interaction and hydrogen bonding, encapsulate self-healing agents and corrosion inhibitors, or assemble to the outside of porous micro/nanocontainers to achieve multilayer wall protection. Common polyelectrolytes include chitosan, sodium alginate, polypyrrole, polyaniline [26], etc. Chitosan is the only polycation in nature that has an ionizable amino group on its molecular chain. These amino groups will be protonated in an acidic environment, thereby changing the permeability of the chitosan layer. By using it as a cationic polyelectrolyte layer material, the microcapsules can be equipped with pH response abilities in an acidic environment. Anionic polyelectrolyte materials and methacrylic acid and its copolymers have excellent film-forming properties and stimulus response capabilities [27], and can provide excellent physical barrier capabilities as anti-corrosion materials.

To prevent further corrosion over time when the pH changes in the coating, it is necessary to add corrosion inhibitors to the core material to inhibit the corrosion process. Débora [28] prepared a new smart coating based on stimulation-responsive microcapsules containing the healing agent linseed oil and the corrosion inhibitor benzotriazole. The coating has two stimulation response mechanisms, mechanical stimulation (release of linseed oil) and pH stimulation (controlled release of benzotriazole), providing self-healing capabilities and inhibiting electrochemical corrosion reactions. Electrochemical impedance spectroscopy (EIS) was used to compare the anti-corrosion properties of microcapsules coated with and without benzotriazole on a carbon steel substrate. The results show that linseed oil alone is not enough to prevent corrosion for a long time, and the self-healing coating containing benzotriazole has a long-term anti-corrosion property. Yun [29] used the self-assembly method to negatively load benzotriazole (BTA) into the calcium carbonate microsphere shell to prepare a new type of highly efficient corrosion inhibition microcapsule, which was added and dispersed into the epoxy resin coating to obtain a new type of corrosion-resistant functional coating. The potentiodynamic polarization curve of aluminum alloy samples soaked in 3.5 wt % NaCl solution with different BTA content for 1 h was tested. The results show that with the increase in BTA content, the self-corrosion potential of aluminum alloy samples increases, and the self-corrosion current density decreases. BTA has a good corrosion inhibition effect on aluminum alloy substrates. The functional coating loaded with BTA inhibitor microcapsules can release BTA in the corrosive medium and effectively protect the substrate. These studies all prove that the addition of BTA enhances the corrosion resistance of the self-healing coating.

Since the corrosion rate of metals such as carbon steel in low-pH environments is much higher than in high-pH environments [30], microcapsules that are sensitive to acidic pH can achieve a more targeted protective effect. Once microcells appear in the coating due to localized corrosion, the microcapsules can selectively release corrosion inhibitors and repair agents where corrosion trends are more severe. Thus, the anti-corrosion ability of the weak parts of the coating is selectively improved, and a new method is provided for the corrosion protection of metals.

Based on the above problems, this paper uses epoxy resin as the coating substrate and uses linseed oil, benzotriazole, and urea–formaldehyde resin to prepare microcapsules through a one-step in situ polymerization method. Chitosan, ethylene glycol dimethacrylate (EGDMA), and α-methacrylic (MA) acid were then used to assemble a polyelectrolyte layer that can respond to changes in acidic pH on the outside of the microcapsule through a layer-by-layer assembly method. An acidic pH-responsive intelligent self-healing coating was prepared using this microcapsule. In addition to responding to mechanical stimulation, it can also respond to local pH changes due to corrosion during the service process of the coating. It can selectively improve the anti-corrosion ability of coatings in low-pH areas and provide a new method for the on-site application of corrosion protection.

## 2. Experimental

### 2.1. Materials

Commercially available Q235 carbon steels were used. Urea (MW:60.6), KBr (MW:119.002), petroleum ether (MW:112), formaldehyde aqueous solution (37 wt%), ammonium chloride (MW:53.49), polyvinyl alcohol 1788 (MW:130.142), acetone (MW:58.08), linseed oil (MW:278.43), hydrochloric acid solution (MW:36.5), absolute ethanol (MW:46.07), α-methacrylic acid (MW:86.09), sodium chloride (MW:58.5), resorcinol (MW:110.11), acetonitrile (MW:41.05), 2,2′-azobisisobutyronitrile (MW:164.208), ethylene glycol dimethacrylate (MW:198.2158), and chitosan (MW:161.16) were purchased from Sinopharm Chemical Reagent Co., Ltd. (Beijing, China). Industrial grade epoxy resin E-44 (MW:180.2_,_ technical grade) and polyamide 650 (technical grade) were purchased from Zhenjiang Danbao Resin Co., Ltd. (Zhenjiang, China). High-purity nitrogen (MW:28.01) was purchased from Keyuan Co., Ltd. (Jinan, China). BYK-A530 defoamer was purchased from BYK Chemicals Co., Ltd. (Berlin, Germany). The phenyltriazole (MW:119.12) was purchased from ALLADDIN. (Los Angeles, CA, USA). All other chemicals used were analytical reagents. All reagents were used as received without further treatment. 

### 2.2. Preparation of Microcapsules

**Figure 1:**
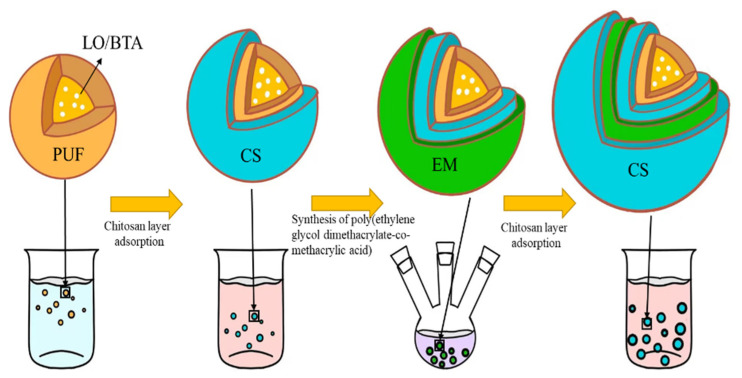
A schematic diagram of the structure and preparation process of the pH-responsive microcapsules.

(1) Preparation of microcapsules: We added 300 mL deionized water, 5 g polyvinyl alcohol solution with a concentration of 5 wt%, 5 g urea, 0.5 g resorcinol, and 0.5 g ammonium chloride into a 500 mL beaker. We used a glass stirring rod to stir the mixture. After all substances were dissolved, the pH of the solution was adjusted to 3.0 using hydrochloric acid at a concentration of 1 wt%. We mixed 20 mL of linseed oil and ground benzotriazole together in an ultrasonic water bath for 10 min to mix the two evenly. The mixture was then poured into a beaker and the solution was mechanically stirred for 10 min at room temperature. When the solution was in a stable state, we slowly heated the solution to 55 °C in a water bath. We slowly added 12.67 g of formaldehyde aqueous solution with a concentration of 37 wt% into the solution and continued stirring at a speed of 500 r/min for 240 min. The suspension obtained by the reaction was cooled to room temperature and washed with deionized water, absolute ethanol, and acetone in sequence, and the prepared microcapsules were collected.
(2) Construct the polyelectrolyte layer: The chitosan clarification solution with a concentration of 1.5 mg/mL was prepared first. We dispersed the microcapsules in 25 mL of chitosan solution with a concentration of 1.5 mg/mL and soaked them at 30 °C for 20 min. We added the microcapsules to a three-neck round-bottomed flask and added 5 mL of acetonitrile and 0.0068 g of α-methacrylic acid. We sealed the three ports of the flask and stirred magnetically at room temperature for 30 min. Afterwards, 5 mg of free radical initiator 2,2′-azobisisobutyronitrile and 0.0495 g of cross-linking agent ethylene glycol dimethacrylate were added to the solution, and stirring was continued for 30 min. The mixture was heated in a water bath at 60 °C for 8 h under a nitrogen atmosphere to form poly(ethylene glycol dimethacrylate-co-methacrylic acid) (EM). After cleaning the microcapsules with deionized water and ethanol, we added 25 mL of chitosan solution and soaked them at 30 °C for 20 min to obtain acidic pH-responsive microcapsules. Figure 1 is a schematic diagram of the process and structure of the microcapsules prepared in this experiment.

### 2.3. Preparation of Acidic pH-Responsive Self-Healing Coatings

Prepare in advance some Q235 carbon steels with dimensions of 25 mm × 50 mm × 2 mm (for scratch experiments, immersion experiments, and salt spray experiments) and 10 mm × 10 mm × 2 mm (for electrochemical experiments). Soak them in absolute ethanol and petroleum ether solution and place them in an ultrasonic water bath for 10 min. Solder wires on the back of the electrochemical sample. Use transparent epoxy resin to encapsulate the sides and back of the sample. After curing, use sandpaper to polish the metal surface. Finally, use deionized water and absolute ethanol to clean the sample surface to obtain a clean sample.

Weigh equal amounts of E-44 epoxy resin and curing agent polyamide 650, add 0.5 wt% defoaming agent BYK-A530 and 10 wt% alcohol, add a certain amount of microcapsules to the epoxy resin, and stir to obtain the microcapsule coating. Uniformly coat the prepared paint and blank epoxy paint on the treated Q235 carbon steel surface with a coater, and coat the side and back of the metal sample. Dry at 25 °C for 24 h.

### 2.4. Characterization

#### 2.4.1. Characterization of Microcapsules

(1) Calculation of encapsulation efficiency and drug loading efficiency

After drying all of the microcapsules obtained in one preparation process, we weighed them with an electronic balance to obtain the total mass of the microcapsules. After grinding, we poured all of the materials into a centrifuge tube and centrifuged them for 5 min. We separated the materials in each layer, added absolute ethanol to extract, dried the bottom wall material, and used Formulas (1) and (2) to calculate the encapsulation rate and drug loading rate of the microcapsules:

(1) $$\mathrm{ER}=\frac{M_{1}-M_{2}}{M_{3}}\times 100\%$$

(2) $$\mathrm{DLR}=\frac{M_{1}-M_{2}}{M_{1}}\times 100\%$$

In the formula, *ER* is the encapsulation rate of the microcapsules, %; *DLR* is the drug loading rate of the microcapsules, %;$\;M_{1}$ is the mass of all microcapsules obtained after drying, g; $M_{2}$ is the mass of the wall material part of all microcapsules obtained after drying, g; $M_{3}$ is the mass of the core material in the preparation raw materials, g.

(2) Test method for sustained release curve

By detecting the release curve of BTA, the changes in the content release of the microcapsules over time in different pH environments were detected.First, the standard solution was obtained by weighing 10 mg BTA and dissolving it into 3.5wt %NaCl solution with pH 5, 7 and 9; we use the standard solution to prepare the concentration of 1, 2, 3, 4, 5, 7, 10, 13, and 15 μg/mL test samples. Using a spectrophotometer (752 N, resolution: 2 nm, transmission) we measured the absorbance at 260 nm [31] to obtain a standard release curve. After that, the microcapsules of the same quality were immersed in a 3.5 wt% sodium chloride solution of pH 5, 7, and 9 to test their absorbance and calculate the concentration of BTA in the medium using the regression equation of the standard curve. The cumulative release rate of BTA in microcapsules under different pH values was calculated according to Formula (3), and the release curve was obtained.

(3) $$\mathrm{RR}=\frac{C_{i}\times V_{S}+\sum_{1}^{\;i-1}C_{j}\times (V_{j}+10)}{M_{S}}\times 100\%$$

In the formula, *RR* is the total release rate of the microcapsules at that time, %; $C_{i}$ is the BTA concentration of the microcapsules at time *i*, μg/mL; $\;V_{S}$ is the total release medium volume, mL; $\;C_{j}$ is the BTA concentration of the microcapsules at time *j*, μg/mL; $\;V_{j}$ is the volume of the release medium taken out at time *j*, mL; $\;M_{S}$ is the fixed total amount of BTA contained in the microcapsules, μg.

(3) Morphological observation

The prepared microcapsules’ size, morphology, and distribution were observed using a three-dimensional confocal concave–convex microscope and a GeminiSEM 300 (Zeiss AG, Baden-Wurtberg, Germany) scanning electron microscope (scanning voltage of 15 kV).

(4) Fourier transform infrared spectroscopy test (FTIR)

After the sample was pressed into potassium bromide tablets, it was scanned with a Nicolet-6700 (Thermo Fisher Scientific, Waltham, MA, USA) Fourier transform infrared spectrometer at 3800~500 cm^−1^.

(5) Thermogravimetric analysis (TGA)

TGA was performed using a TGA/DSC 3+ thermogravimetric analyzer (METTLER TOLEDO, Zurich, Switzerland). The test conditions were as follows: using air as the reference gas, in a nitrogen environment, the microcapsules were slowly heated from 30 °C to 600 °C at a speed of 10 °C/min.

(6) Zeta potential test

We used the SurPass 3 Zeta surface potential analyzer (AntonPaar, Austria) to test the zeta potential of the microcapsules during the preparation process and recorded the value after diluting the microcapsules 1000 times in deionized water. The test environment pH was 7.

#### 2.4.2. Characterization of Self-Healing Properties of Coatings

We used a #11 scalpel blade to make an “X-shape” scratch on the coated surface. Scratch depth and coating thickness were measured using a three-dimensional confocal concave–convex microscope.

We placed the coating sample with scratches on the surface in air and let it stand for 24 h. We calculated the repair rate of the scratch depth through Formula (4):

(4) $$\mathrm{RPR}=\frac{H_{0}-H_{24}}{H_{0}}\times 100\%$$

In the formula, RPR is the repair rate of the scratch depth,$\;H_{0}$ is the scratch depth at the initial time, and $H_{24}$ is the scratch depth detected 24 h after the scratch.

#### 2.4.3. Characterization of Anti-Corrosion Properties of Coatings

(1) Salt spray test

We placed the sample into a salt spray box with the scratched side facing up at an angle of 30° to the ground and exposed it to a neutral (pH = 6.5~7.2) or acidic (pH = 5) salt spray environment. The temperature inside the salt spray box was 35 °C, the salt spray solution used was a 3.5 wt% sodium chloride solution, and the average collection rate of the salt spray solution was 1.4 mL/h [32].

(2) Electrochemical testing

After scratching the prepared coating sample using a scratching tool, the electrochemical impedance spectrum of the sample was tested using the PARSTAT 2273 electrochemical workstation. The experiment adopted a three-electrode system, in which the auxiliary electrode was a platinum electrode, the reference electrode was a saturated calomel electrode, the working electrode was a coated sample, and the composition of the solution used for the electrochemical test was a 3.5 wt% NaCl solution. The test process used a frequency range of 10^−2^~10^5^ Hz and an AC sine wave amplitude of 10 mV. A total of 60 points were tested in each group.

## 3. Results and Discussion

### 3.1. Optimization of Microcapsule Preparation Process

#### 3.1.1. Effect of Chitosan Concentration on Microcapsules

**Figure 2:**
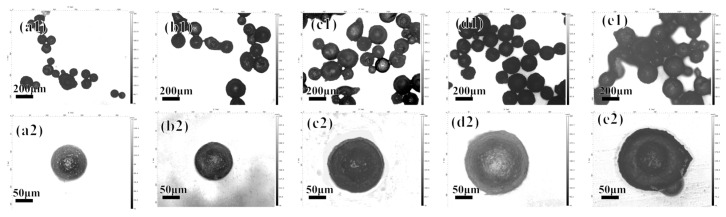
The morphology of the acidic pH-responsive microcapsules prepared at chitosan concentrations of 0.5, 1, 1.5, 2, and 2.5 mg/mL shown under a 3D confocal microscope: (**a1**–**e1**) 5× magnification; (**a2**–**e2**) 20× magnification.

When other parameters remain unchanged, the concentration of CS is changed to 0.5, 1, 1.5, 2, and 2.5 mg/mL, and the results are shown in Figure 2.

Obviously, within the concentration range of 0.5~2 mg/mL, the particle size of the microcapsules continues to increase, and the surface is also smoother. This is because when the concentration of chitosan increases, the microcapsules carry a larger amount of positive charges, which can attract more oppositely charged polymers to deposit and form a smooth surface. However, when the concentration is increased to 2.5 mg/mL, the microcapsules show obvious adhesion. This is because the last layer of the layer-by-layer assembly is chitosan, which itself is highly sticky and insoluble in the microcapsule preparation conditions.

**Figure 3:**
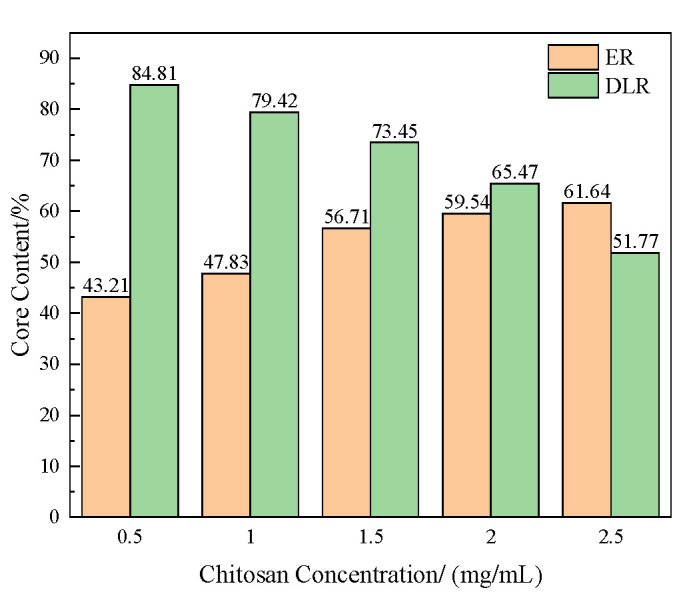
The encapsulation rate and drug loading rate of the acidic pH-responsive microcapsules prepared when the concentration of chitosan is 0.5, 1, 1.5, 2, and 2.5 mg/mL.

The encapsulation rate and drug loading rate of several prepared microcapsules were tested. The method is shown in Section 2.4.1. The results are shown in Figure 3.

It can be seen that as the concentration of chitosan increases, the encapsulation rate of microcapsules shows a trend of increasing first quickly and then slowly, while the drug loading rate continues to decrease. This is because when the chitosan concentration is low, there is less positively charged chitosan attached to the microcapsules, making layer-by-layer assembly difficult. The resulting microcapsule wall layer is overall thin and easily damaged. When the chitosan concentration is too high, the stability of the microcapsules increases to the upper limit, but the wall thickness continues to increase, and the core-to-wall ratio is too low.

**Figure 4:**
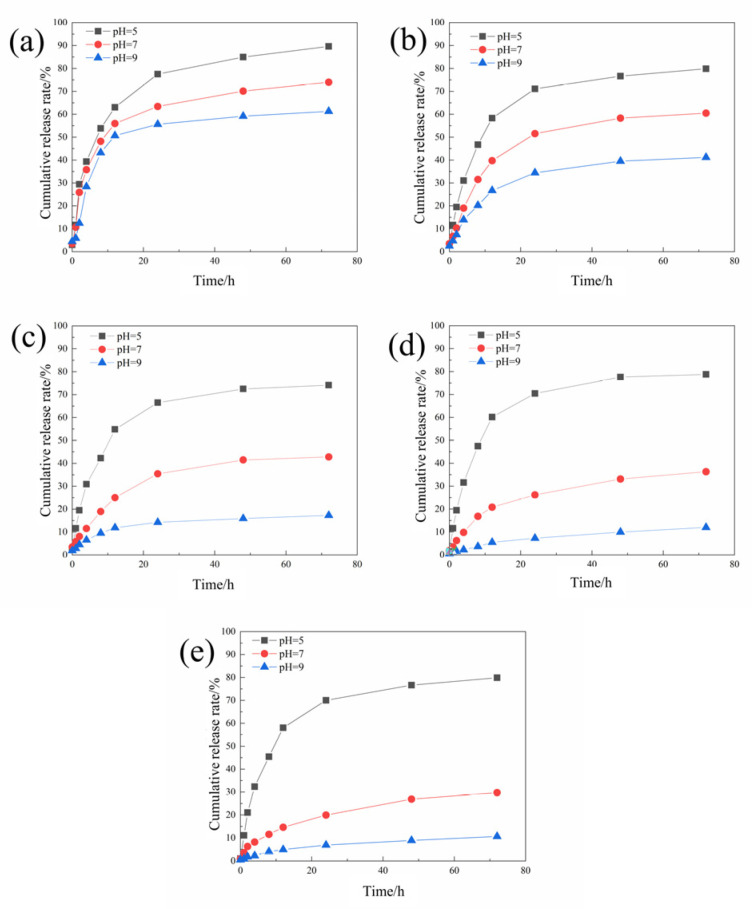
The sustained release curves of the acidic pH-responsive microcapsules prepared when the chitosan concentrations were 0.5 (**a**), 1 (**b**), 1.5 (**c**), 2 (**d**), and 2.5 (**e**) mg/mL.

The sustained release curves of different microcapsules were tested according to the method in Section 2.4.1, and the results are shown in Figure 4.

The cumulative release rate of the microcapsules decreases with the increase in the pH value. This is because when the environment is acidic, chitosan will be protonated, causing electrostatic repulsion between the polymers to become loose, allowing the core material to be released through the pores. As the concentration of chitosan continues to increase, the microcapsules become more sensitive to pH stimulation.

It can be found that although increasing the chitosan concentration is beneficial to improving the pH-responsiveness and encapsulation efficiency of the microcapsules, when the chitosan concentration is too high, the morphology and drug loading rate of the microcapsules has deteriorated. Therefore, 2 mg/mL was selected as the optimal chitosan concentration for preparing microcapsules.

#### 3.1.2. Effect of Polymerization Time on Microcapsules

**Figure 5:**
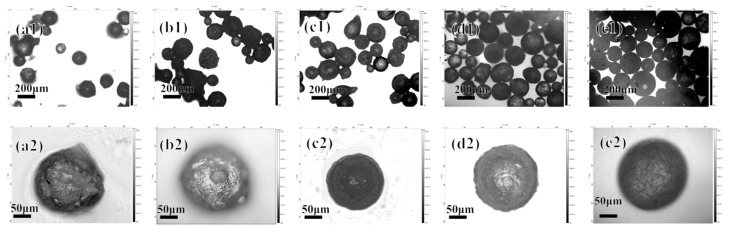
The morphology of the acidic pH-responsive microcapsules prepared at polymerization times of 2, 4, 6, 8, and 10 h shown under a 3D confocal microscope: (**a1**–**e1**) 5× magnification; (**a2**–**e2**) 20× magnification.

Assembling the polyelectrolyte layer requires cross-linking through polymerization reactions to form negatively charged EM. When other parameters remain unchanged, the polymerization time is changed to 2 h, 4 h, 6 h, 8 h, and 10 h, and the morphology of the prepared microcapsules is shown in Figure 5.

It can be seen that in the range from 2 to 8 h, the polymerization time has a greater impact on the morphology of the microcapsules. When the polymerization time is short, the particle size distribution of the microcapsules is uneven, and there are a large number of adhesions and lumps on the surface (Figure 5a,b). This is due to the inability of the polymer produced during the reaction to completely encapsulate all of the microcapsules. At the same time, because the surface of the microcapsules does not have sufficient negative charges, excess chitosan precipitates during the layer-by-layer assembly process. As the polymerization time increases, the sphericity and dispersion of the microcapsules are good, which is due to the cross-linking of α-methacrylic acid on the surface of the microcapsules to form a dense polymer layer.

**Figure 6:**
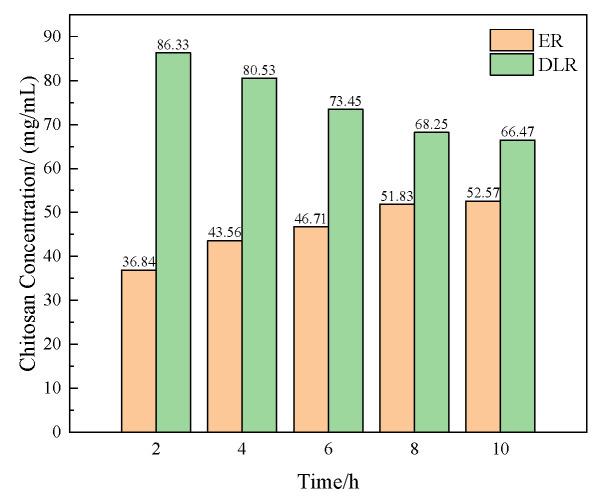
The coating rate and drug loading rate of the acidic pH-responsive microcapsules prepared when the polymerization time is 2, 4, 6, 8, and 10 h.

**Figure 7:**
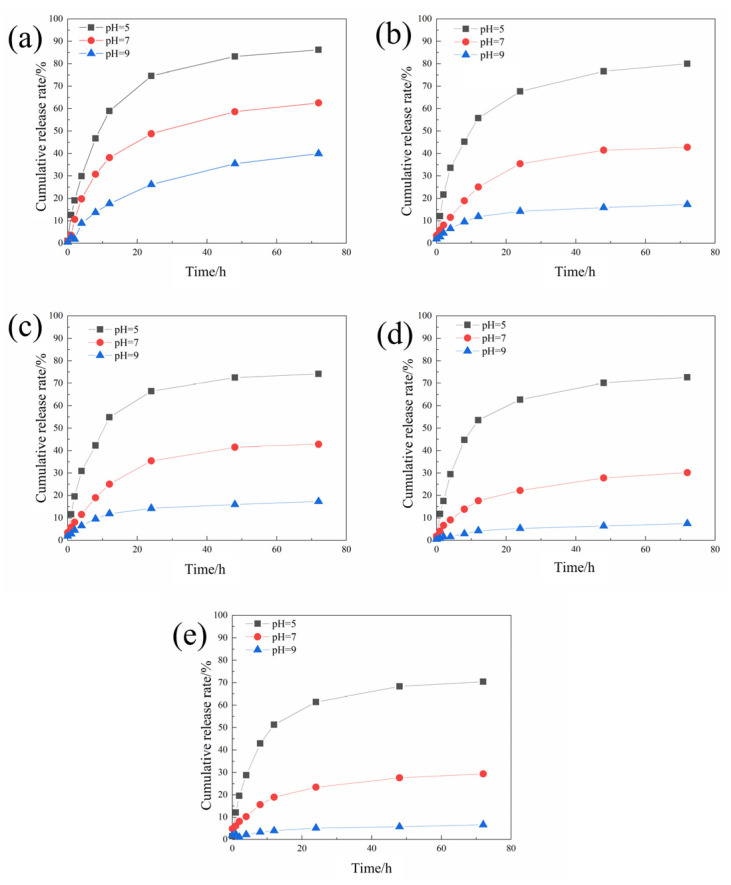
The sustained release curves of the acidic pH-responsive microcapsules prepared when the polymerization time is 2 (**a**), 4 (**b**), 6 (**c**), 8 (**d**), and 10 (**e**) h.

According to the method described in Section 2.4.1, several prepared microcapsules were tested for encapsulation rate, drug loading rate, and sustained release curve. The results are shown in Figure 6 and Figure 7.

With the extension of polymerization time, the coating rate of microcapsules increased continuously, the drug loading rate decreased continuously, and the microcapsules had more obvious pH response. The analytical reason is that when the polymerization time is too low, the microcapsules do not have sufficient strength. As the polymerization time increases, the polymer layer successfully forms a denser and high-thickness multilayer wall structure, but the core-to-wall ratio decreases.

By comparison, it can be found that when the polymerization time reaches 10 h, there is not much change overall. This shows that cross-linking has been basically completed in 8 h, and continuing to increase the heating time will only increase the preparation time and cost. Taking the above results into consideration, 8 h was finally selected as the optimal polymerization time for preparing microcapsules.

#### 3.1.3. Effect of Heating Temperature during Polymerization on Microcapsules

**Figure 8:**
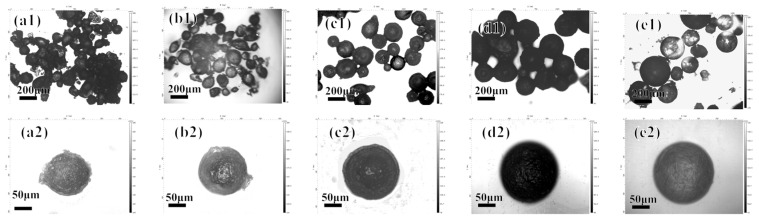
The morphology of the acidic pH-responsive microcapsules prepared at heating temperatures of 30, 45, 60, 75, and 90 °C shown under a 3D confocal microscope: (**a1**–**e1**) 5× magnification; (**a2**–**e2**) 20× magnification.

The properties and morphology of microcapsules are also affected by the temperature of the polymerization process [31]. With other parameters unchanged, the heating temperature of the polymerization process was changed to 30, 45, 60, 75, and 90 °C. The morphology of the prepared microcapsules is shown in Figure 8.

It can be seen that when the temperature is too low (Figure 8(a1,b1)) or too high (Figure 8(e1)), excess impurities are produced. This is because when the polymerization temperature is too low, the initiator decomposes slowly and the number of free radicals in the solution is small. It is difficult for monomers to polymerize into a completely negatively charged polymer layer, and the microcapsules have poor stability. If the temperature is too high, the free radicals in the solution react rapidly with the monomers, forming excess macromolecular free radicals. Some macromolecular free radicals react with each other to form stable polymers, which then adsorb chitosan to form polyelectrolyte particles.

**Figure 9:**
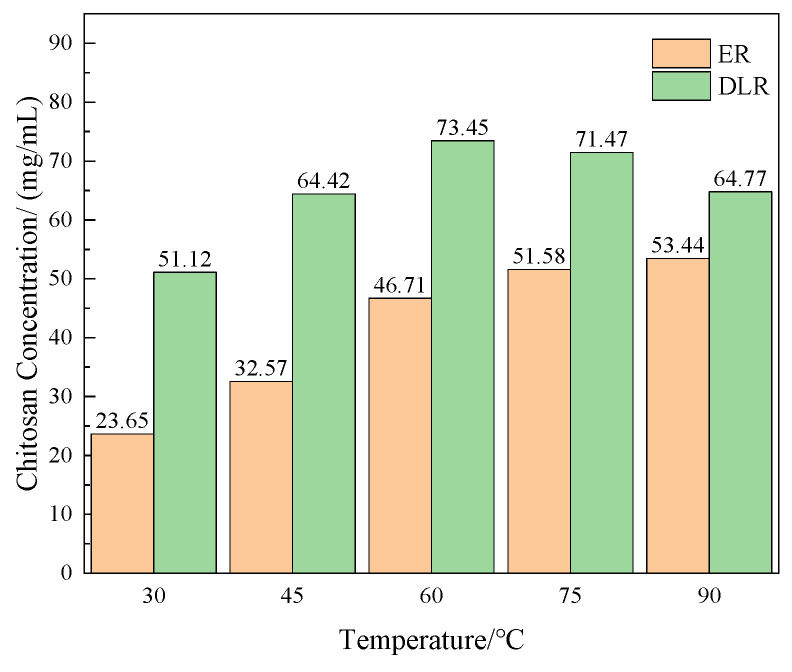
Coating rate and drug loading rate of acidic pH-responsive microcapsules prepared at heating temperatures of 30, 45, 60, 75, and 90 °C.

**Figure 10:**
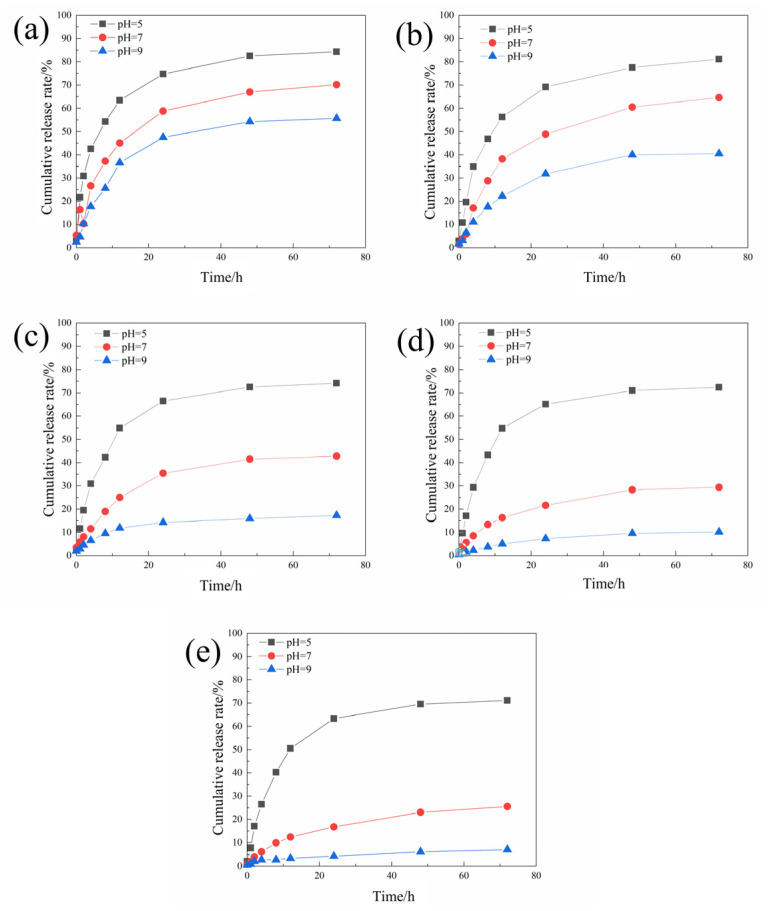
Sustained release curves of acidic pH-responsive microcapsules prepared at heating temperatures of 30 (**a**), 45 (**b**), 60 (**c**), 75 (**d**), and 90 (**e**) °C.

Several prepared microcapsules were tested for encapsulation rate, drug loading rate, and sustained release curve using the method in Section 2.4.1. The results are shown in Figure 9 and Figure 10.

It can be found that the pH stimulus responsiveness and encapsulation rate of the microcapsules first increase quickly and then slowly as the temperature increases, while the drug loading rate shows a trend of first increasing and then decreasing. The reason is that when the temperature is too low, the microcapsules cannot assemble multilayered dense film layers through the polymerization reaction, and do not have good stability in alkaline and neutral solutions, making it difficult to protect the core material. When the temperature is too high, many polyelectrolyte particles are produced in the solution, and the product is no longer pure. At 75 °C, the rate is sufficient for the polymerization reaction to be completed, and the outer wall of each microcapsule is stable enough, so the encapsulation efficiency and sustained release curve no longer change significantly after the temperature is raised.

Taking the above results into consideration, 75 °C was finally selected as the optimal heating temperature for microcapsules.

#### 3.1.4. Effect of Number of Polyelectrolyte Layers on Microcapsules

**Figure 11:**
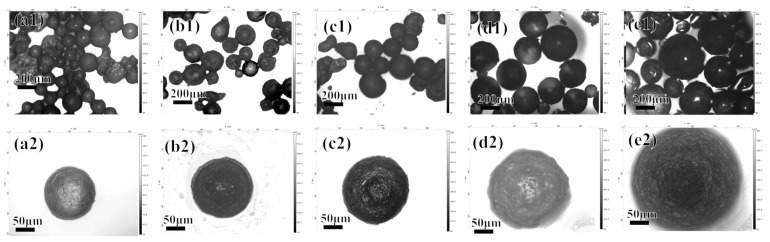
The morphology of the acidic pH-responsive microcapsules prepared when the number of polyelectrolyte layers is 1, 3, 5, 7, and 9 shown under a 3D confocal microscope: (**a1**–**e1**) 5× magnification; (**a2**–**e2**) 20× magnification.

The sustained release performance of a microcapsule is closely related to the number of polyelectrolyte layers assembled on its surface. In this study, we continued to assemble different layers of negatively charged polymer EM and positively charged chitosan on the outer layer of the microcapsules. While other parameters remained unchanged, the number of polymerization layers was changed to 1, 3, 5, 7, AND 9. The morphology of the prepared microcapsules is shown in Figure 11.

It is obvious that as the number of polyelectrolyte layers increases, the particle size of the microcapsules increases significantly, reaching 103, 150, 174, 277, and 378 μm, respectively, and the surface becomes smoother and smoother. Since the increase in the number of layers will greatly increase the time and cost of preparation, and microcapsules with too large particle sizes may affect the bonding force and mechanical strength of the coating, microcapsules with too many layers are not suitable for use on site.

**Figure 12:**
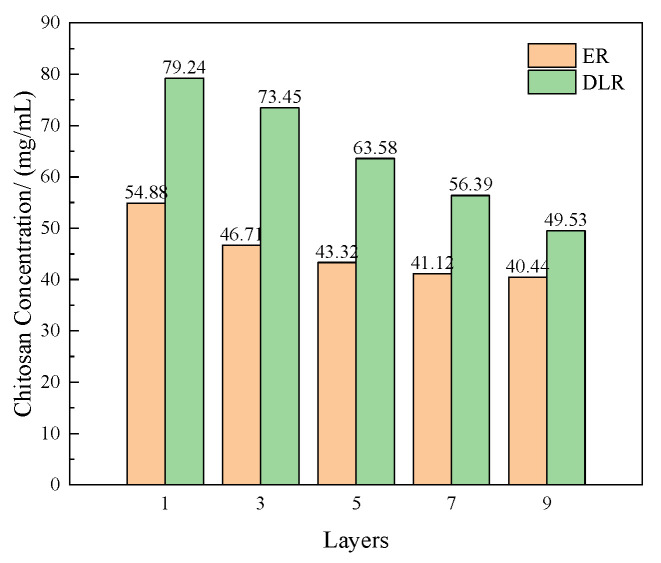
The coating rate and drug loading rate of the acidic pH-responsive microcapsules prepared when the number of polyelectrolyte layers is 1, 3, 5, 7, and 9.

**Figure 13:**
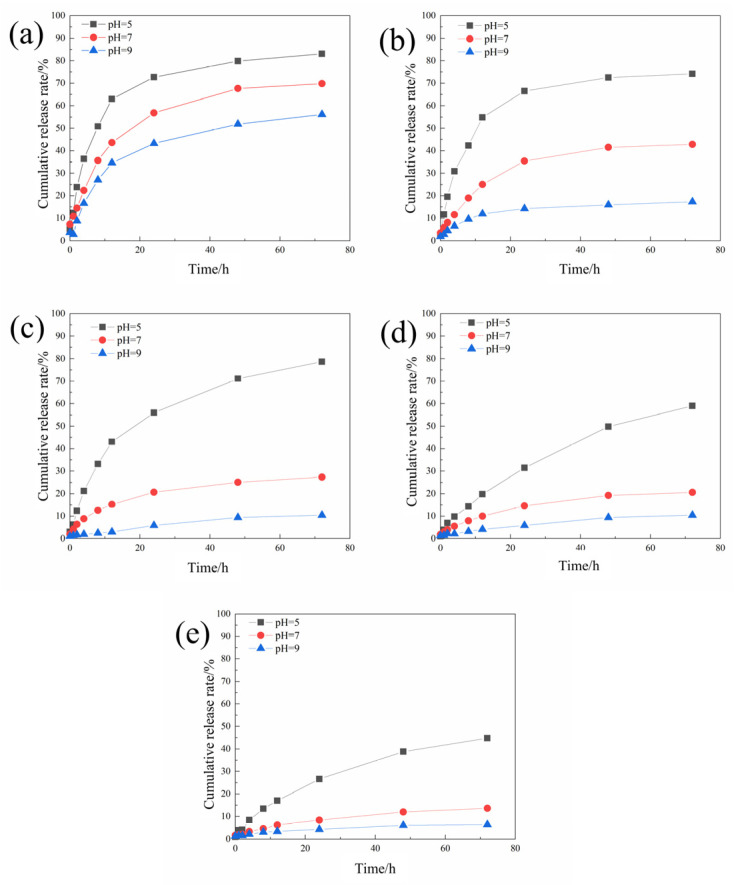
The sustained release curves of the acidic pH-responsive microcapsules prepared when the number of polyelectrolyte layers is 1 (**a**), 3 (**b**), 5 (**c**), 7 (**d**), and 9 (**e**).

Several prepared microcapsules were tested for encapsulation rate, drug loading rate, and sustained release curve. The method is shown in Section 2.4.1. The results are shown in Figure 12 and Figure 13.

It can be found that as the number of layers increases, the encapsulation rate and drug loading rate of the microcapsules show a gradually decreasing trend. The reason is that as the layer-by-layer assembly continues, the microcapsules undergo multiple stirrings and filtrations. When the number of layers is low, these mechanical stimuli destroy many microcapsules. As the number of layers increases, the wall thickness of the microcapsules increases, making it more difficult to destroy, and the core-to-wall ratio becomes smaller.

The stimulus response sensitivity of the microcapsules first increases and then decreases. When there is only one layer, the release rate of the microcapsules is very high in all three environments. This is because the wall layer is too thin and the stability of the microcapsules is insufficient. After increasing the number of layers to three, the microcapsules have obvious acidic stimulus responsiveness. When the number of layers is too high, the release of microcapsules under acidic conditions becomes slow. The analytical reason is that as the number of layers increases, it takes longer for hydrogen ions to change the permeability of the innermost layer.

Based on the above results, the number of polyelectrolyte layers was finally selected as three for the preparation parameters of microcapsules.

### 3.2. Properties of Microcapsules

#### 3.2.1. Microcapsule Morphology and Sustained Release Curve

After optimization, the microcapsules have higher sphericity, a smooth surface, and good dispersion, and the average particle size increases to 203 μm. The test shows that the encapsulation rate of the optimized microcapsules is 59.74% and the drug loading rate is 63.99%.

**Figure 14:**
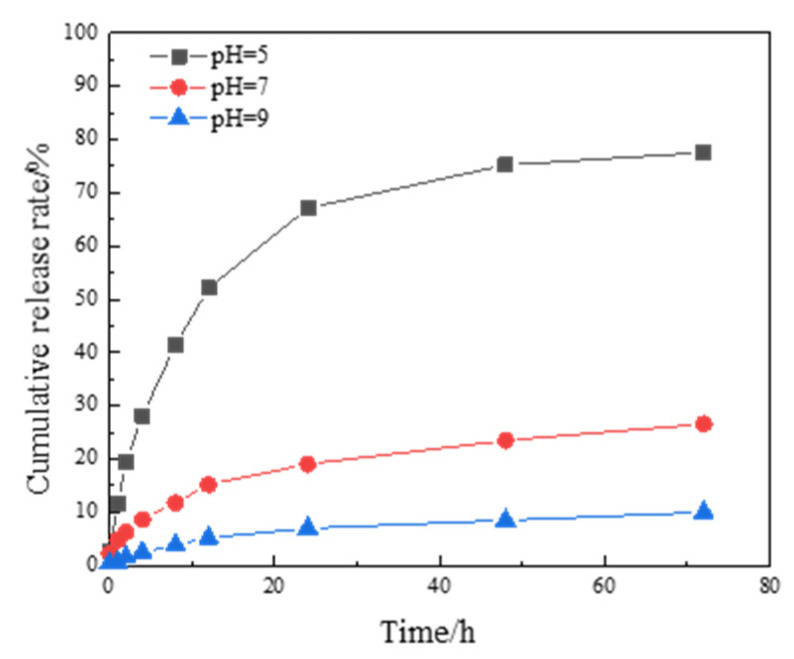
Optimized sustained release curve of acidic pH-responsive microcapsules.

The sustained release curve of the optimized microcapsules was tested using a UV–visible spectrophotometer, as shown in Figure 14.

The optimized microcapsules exhibit obvious stimulus responsiveness. They have a fast response speed in acidic environments and strong stability in neutral and alkaline environments, which can avoid releasing too much content before corrosion occurs in the coating.

**Figure 15:**
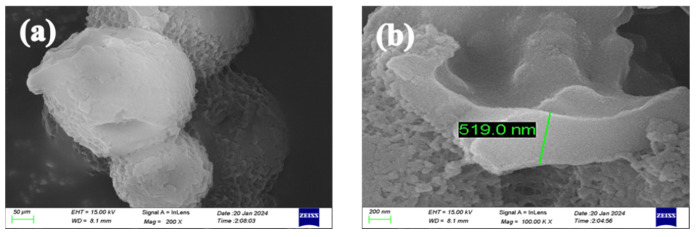
SEM of optimized acidic pH-responsive microcapsules: (**a**) surface of microcapsule; (**b**) section after microcapsule rupture.

The prepared acidic pH-responsive microcapsules were observed using a scanning electron microscope. Figure 15a shows the surface morphology, and Figure 15b shows the cross-section of the microcapsule after rupture. It can be seen that the shell thickness at the cross-section is about 519 nm.

#### 3.2.2. FTIR

**Figure 16:**
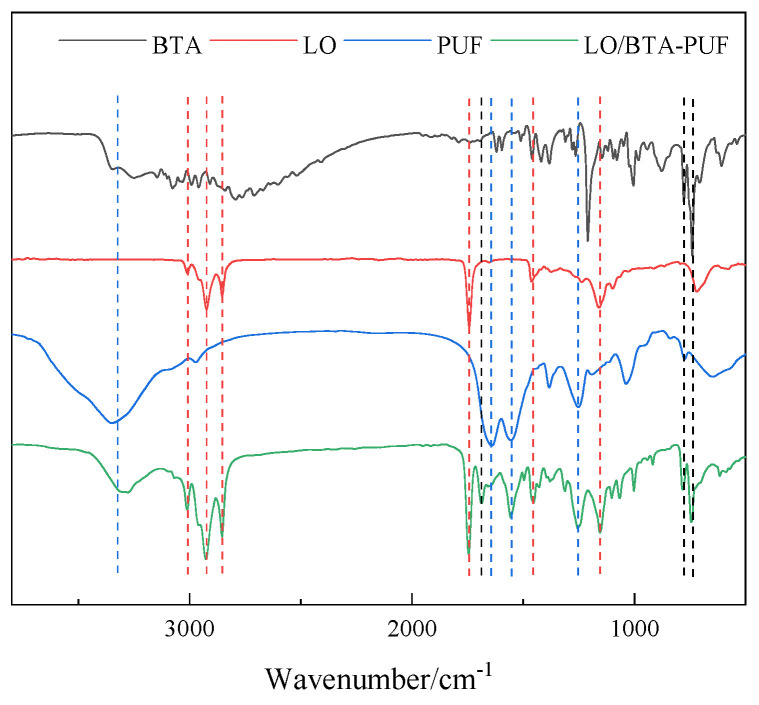
FTIR spectra of urea−formaldehyde resin microspheres (black curve), linseed oil (red curve), benzotriazole (blue curve), and LO/BTA−PUF (green curve).

To confirm the successful generation of microcapsules (LO/BTA-PUF), urea-formaldehyde resin microspheres, linseed oil, benzotriazole, and LO/BTA-PUF were tested using the method described in Section 2.4.1. The results are shown in Figure 16.

It can be found that four characteristic peaks at 3325.212 cm^−1^, 1649.804 cm^−1^, 1559.166 cm^−1^, and 1251.641 cm^−1^ on LO/BTA-PUF in Figure 16 correspond to several characteristic peaks of urea–formaldehyde resin microspheres. They are, respectively, attributed to the stretching vibration absorption peak after the –O–H bond in the hydroxymethyl group overlaps with the –N–H bond in the amide group, the stretching vibration peak of the C=O bond in the secondary amide group, the stretching vibration absorption peak of C–N in the amide bond, and the –C–H saturated bond stretching vibration absorption peak, which indicates that the polyurea–formaldehyde wall material was successfully synthesized using the in situ polymerization method. It can be seen in the FTIR spectrum of linseed oil that it has three obvious characteristic peaks at 1745.212 cm^−1^, 1456.292 cm^−1^, and 1164.363 cm^−1^, and three absorption peaks at 2854.614 cm^−1^, 2929.480 cm^−1^, and 3010.738 cm^−1^. They are attributed to the C=O stretching and C–O stretching vibration peaks and the stretching and bending vibrations of sp^3^C–H bonds, respectively. At the same time, two obvious characteristic peaks appear at position 748.290 cm^−1^ and 790.424 cm^−1^ in the FTIR spectrum of benzotriazole which are attributed to the ortho-disubstituted peak of the benzene ring and the C–H bending vibration peak of the benzene ring. These characteristic peaks can all correspond to the characteristic peaks on LO/BTA-PUF. This shows that the core materials BTA and LO were successfully encapsulated into microcapsules.

**Figure 17:**
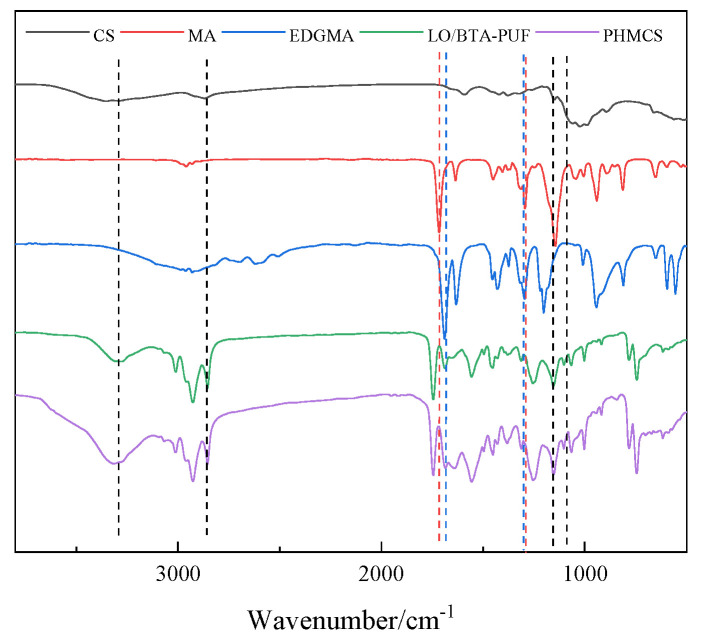
FTIR spectra of CS (black curve), MA (red curve), EDGMA (blue curve), LO/BTA−PUF (green curve), and pHMCSs (purple curve).

To verify the successful assembly of the polyelectrolyte layer, the functional groups of CS, MA, EDGMA, LO/BTA-PU, and the acidic pH-responsive microcapsules (PHMCSs) were characterized, as shown in Figure 17.

It can be seen that chitosan has a broad and large characteristic peak at around 3313.201 cm^−1^. This can be attributed to the N–H and O–H stretching peaks of chitosan and the intramolecular hydrogen bonds. The strong vibration peak at 2838.440 cm^−1^ corresponds to the C–H symmetric and asymmetric stretching vibration peaks of chitosan. The characteristic peaks at 1089.584 cm^−1^ and 1154.188 cm^−1^ correspond to the C–O stretching peak and C–O–C stretching peak of chitosan. Comparing the FTIR spectra of LO/BTA-PUF and the PHMCSs, it can be found that the peak intensity in the corresponding region has increased, which indicates the successful assembly of chitosan. In addition, it can be seen that in the FTIR spectra of MA and EDGMA, there are two obvious characteristic peaks at 1292.072 cm^−1^ and 1285.805 cm^−1^. These can be attributed to the stretching vibration peaks of C-O bonds in the symmetric and asymmetric esters of EGDMA with MA, respectively. At the same time, it can be seen that there are two obvious characteristic peaks in the range from 1682.492 cm^−1^ to 1738.031 cm^−1^ which are due to the stretching vibration peak of the carbonyl group contained in EGDMA and MA. Although no obvious characteristic peak appears in the FTIR spectrum of the pH-responsive microcapsules, their peak intensity is higher than that of LO/BTA-PUF. This demonstrates the successful assembly of the EM layer. In summary, it can be demonstrated that the polyelectrolyte layer was successfully assembled on the outside of each microcapsule.

#### 3.2.3. TGA

**Figure 18:**
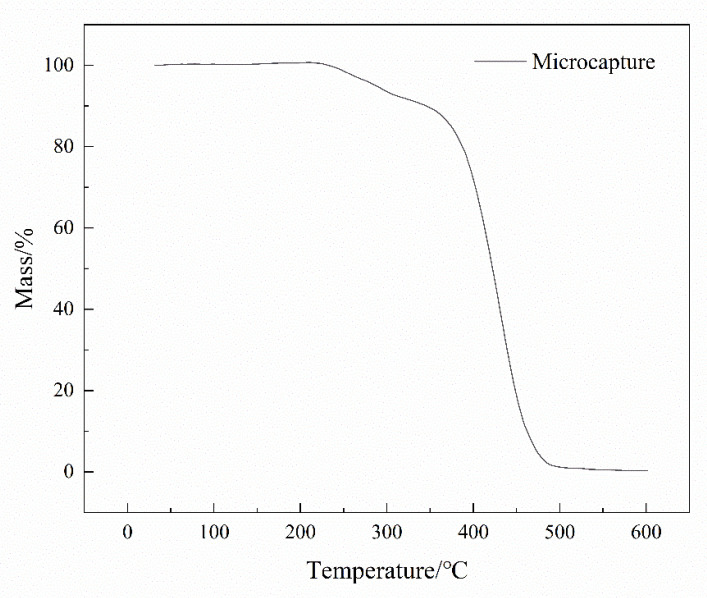
TGA curves of pH-responsive microcapsules.

Good thermal stability is conducive to extending the service life of the coating and expanding the application field of the coating. This study tested the thermal stability of the acidic pH-responsive microcapsules through the method described in Section 2.4.1. The TGA curve obtained by the test is shown in Figure 18.

BTA has a melting point of 98 °C and a boiling point of 204 °C at standard atmospheric pressure [33]. It can be seen from the figure that the microcapsules have sufficient stability at 30~220 °C, and the first weight loss is only 0.22%. This loss is mainly due to water evaporation and interlayer water loss during the detection process. When the temperature rises from 220 °C to 380 °C, the microcapsules begin to slowly decompose, losing a total of 17.5% of their weight at this stage. This is related to the decomposition of the outer polyelectrolyte shell of the microcapsules. At the stage of 380~500 °C, the mass loss of the microcapsules is very severe, reaching 82.4%. The reason is that the wall layer of the microcapsules is completely decomposed by heat [34] and no longer has a good protective effect. The core materials linseed oil and benzotriazole are decomposed in large quantities at high temperatures, and the final mass loss is greatly increased. According to the overall trend of the TGA curve in Figure 18, it can be considered that the thermal decomposition temperature of the microcapsules is 220 °C. Because the microcapsules need to be added to the epoxy resin, when the epoxy resin is used in air, the thermal oxidation decomposition temperature is usually around 180~200 °C, which is lower than the thermal decomposition temperature of the microcapsules. Therefore, the thermal stability of the microcapsules can meet the basic needs of the anti-corrosion coating application environment.

#### 3.2.4. Zeta Potential Test

**Figure 19:**
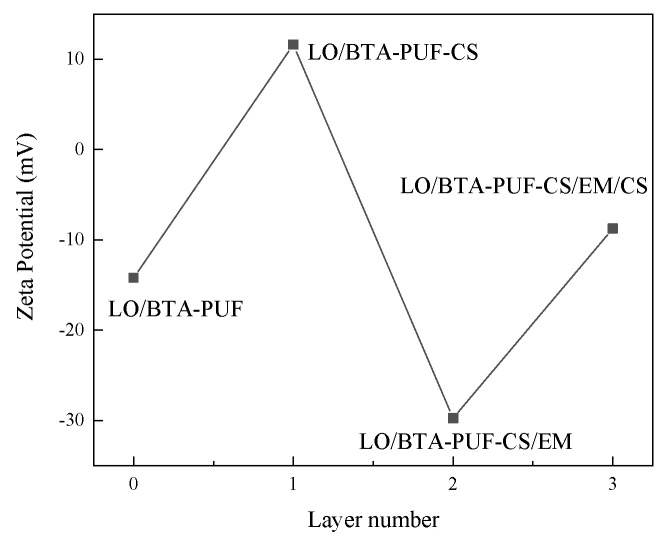
Zeta potential diagram of microcapsules during layer−by−layer assembly.

Zeta potential testing can effectively verify the assembly of the polyelectrolyte layer on the surface of microcapsules. The surface charge of LO/BTA-PUF and the multilayer microcapsules obtained after assembling each layer of polymer layer by layer was studied through zeta potential testing. The results are shown in Figure 19. The initial zeta potential of LO/BTA-PUF is −14.2 mV. After adsorbing the polycationic layer CS, the zeta potential of the microcapsules increases significantly, reaching +11.6 mV, achieving charge reversal. The second layer of polyanion-layer EM adsorbed on the microcapsule causes the zeta potential of the microcapsule to significantly decrease to −29.75 mV. After assembling a layer of CS on the outside of the microcapsule again through the layer-by-layer assembly method, it can be seen that the zeta potential of the microcapsule is smaller than the negative value of the previous layer. This proves the adsorption of the positive layer, as part of the positive charge compensates for the negative charge of the previous layer, and the final zeta potential of the microcapsule surface is −8.75 mV. The illustrated results demonstrate the layer-by-layer assembly of multilayer polyelectrolyte layers on the outside of the microcapsules.

### 3.3. Self-Healing Properties of Coatings

**Figure 20:**
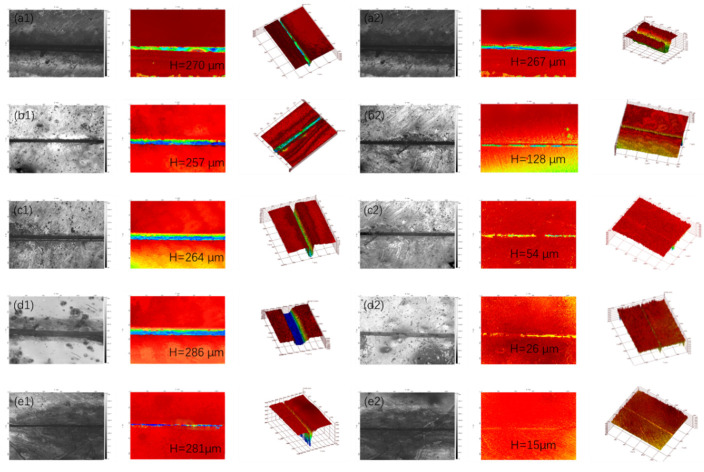
The scratch changes of samples containing 0 wt%, 5 wt%, 10 wt%, 15 wt%, and 20 wt% acidic pH-responsive microcapsules before and after repair: (**a1**–**e1**) before repair; (**a2**–**e2**) after repair. Different colors correspond to different heights of coatings.

The thickness of the coating obtained by the three-dimensional confocal concave–convex microscope test in Section 2.4.2 ranges from 260 to 270 mm. Figure 20 shows the scratch changes of samples with 0 wt.%, 5 wt%, 10 wt%, 15 wt%, and 20 wt% acidic pH-response microcapsules observed using a 3D confocal microscope before and after standing. Among them, there are three maps in each group which are divided into a two-dimensional topography map (left), a height distribution map (middle), and a three-dimensional topography map (right). The colors in the height distribution map correspond to the height of the coating surface. To better quantify the self-healing effect induced by the microcapsules, the repair rate RPR of the scratch depth is used as an index to quantitatively evaluate the repair effect of the coating.

The calculated RPR of the coating samples containing 0 wt%, 5 wt%, 10 wt%, 15 wt%, and 20 wt% acidic pH-responsive microcapsules was 1.11%, 50.19%, 79.54%, 90.90%, and 94.66%, respectively. It can be found that within the range from 0 wt% to 20 wt%, the scratch repair rate is significantly positively correlated with the amount of microcapsules added. The reason is that microcapsules containing healing agents are embedded into the coating material. When microcracks appear in the coating, the microcapsules will be affected and crack, and the healing agents in the middle will fill the cracks through the capillary mechanism and repair the damaged part through polymerization or oxidation to achieve the purpose of healing. With the increase in microcapsule addition, the probability of microcapsule rupture triggered by crack increases. More healing agents are released and scratches are repaired better. The experimental results show that when the microcapsule content is 15 wt% and above, the coating has good self-healing properties.

### 3.4. Anti-Corrosion Properties of Coating

#### 3.4.1. Research on the Anti-Corrosion Performance of the Self-Healing Coating Based on the Salt Spray Test

**Figure 21:**
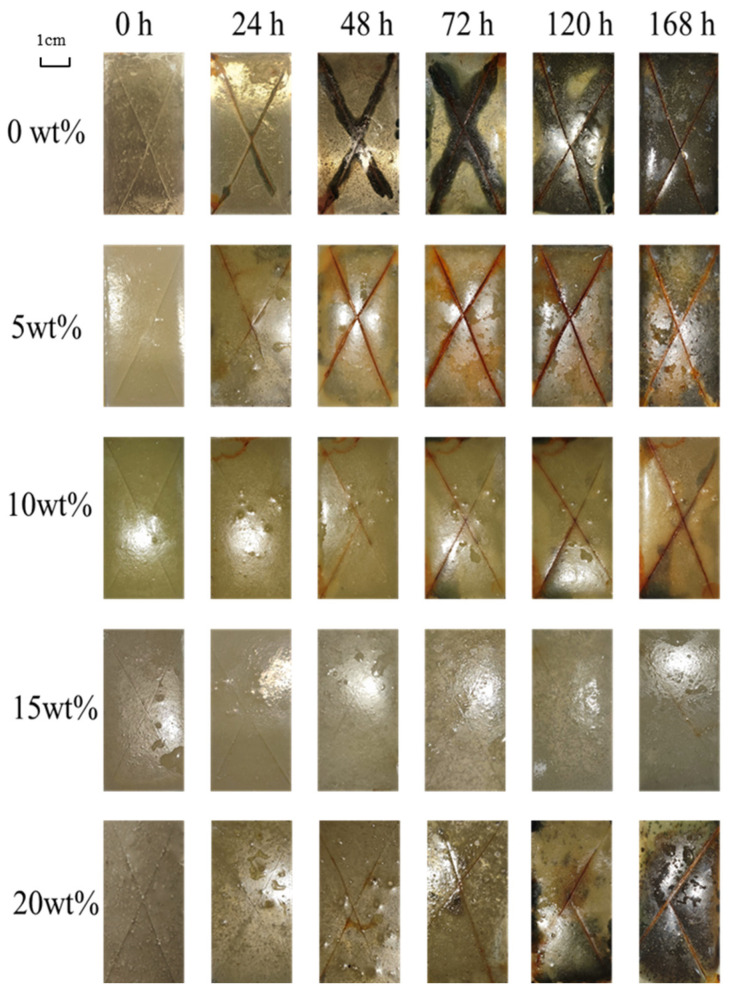
Morphology of coatings with different microcapsule contents during salt spray test.

This paper conducted neutral salt spray experiments on coating samples containing 0 wt%, 5 wt%, 10 wt%, 15 wt%, and 20 wt% acidic pH-responsive microcapsules. The specific test methods are described in Section 2.4.3, and the results are shown in Figure 21.

It can be seen from Figure 21 that the amount of microcapsules added has a very obvious impact on the anti-corrosion performance of the coating. Among them, the corrosion of pure epoxy samples is the most severe. This is because the pure epoxy coating does not have a self-healing effect. In the harsh environment of the salt spray experiment, the high-temperature environment in the box causes water mist to penetrate under the film and continue to spread, reducing the bonding force between the coating and the metal.

After adding microcapsules, the anti-corrosion performance of the coating is improved. When a small amount of microcapsules is added, the anti-corrosion effect is limited, and a large number of black corrosion marks still appear in the later stages of the experiment. As the amount of microcapsules added increases, the anti-corrosion properties of the coating further increase. When the addition amount reaches 15 wt%, there are still no corrosion marks on the surface of the coating after 120 h of the experiment, and no edge corrosion occurs during the entire experiment period.

However, when the additional amount continues to increase to 20 wt%, bubbling and spot corrosion appear inside the coating. After 168 h, the coating completely separates from the metal substrate, and the corrosion is extremely severe. This is due to the excessive addition of microcapsules, which introduces too many defects into the epoxy coating and affects the shielding performance of the coating.

**Figure 22:**
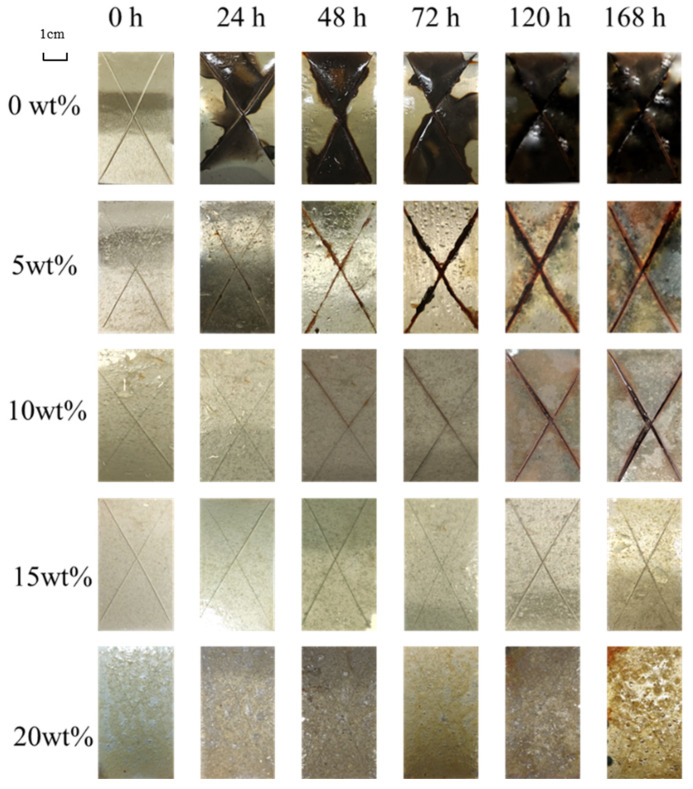
Morphology of coatings with different microcapsule contents during acidic salt spray test.

To verify the pH-responsiveness of the microcapsules, the difference in the anti-corrosion ability of the coating in an acidic salt spray environment was tested. The results are shown in Figure 22.

It can be seen that in an acidic environment, the corrosion rate of the pure epoxy coating is significantly faster than that in a neutral environment. The reason is that the corrosion rate is accelerated at low pH and the coating fails quickly.

Compared with the experimental results in a neutral salt spray environment, in an acidic environment, no obvious corrosion occurred in the unscratched position of the coating, and the overall corrosion rate was also lower than that in a neutral environment. The reason is that in an acidic environment, the microcapsules in non-scratch areas are stimulated by pH changes to release the core material, thereby improving the shielding performance of the coating.

In addition, the coating with too many microcapsules still showed slight corrosion after 168 h. The reason is that excessive filler and broken shells during the preparation process still harms the physical properties of the coating. There are still some pores in the coating that have become the weak points of the coating.

Based on the above experimental results, it can be found that when the additional amount of microcapsules is 15 wt%, the coating has the best anti-corrosion performance.

**Figure 23:**
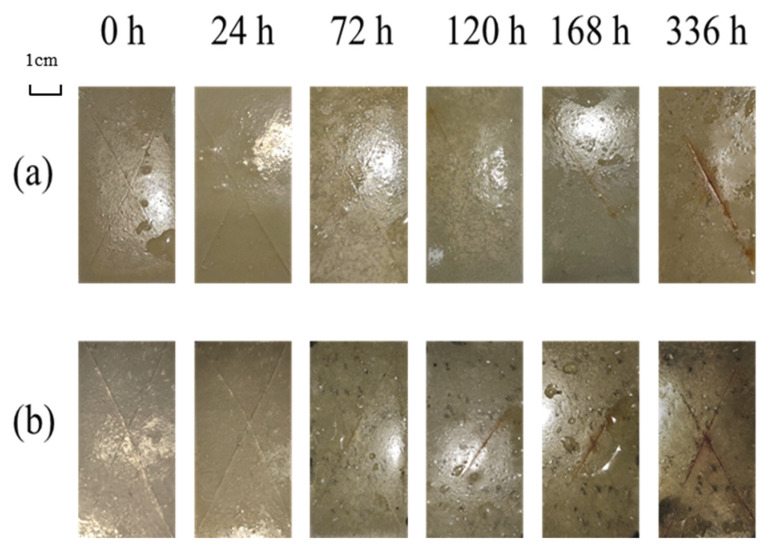
The morphology of different types of microcapsule coatings during the salt spray test: (**a**) the acidic pH-responsive intelligent self-healing coating; (**b**) the ordinary self-healing coating.

To explore the difference between acidic pH-responsive self-healing anti-corrosion coatings and ordinary self-healing coatings, coatings with the same microcapsule content were prepared and tested in long-cycle salt spray experiments. The test duration was 336 h in total, and the samples were taken out for observation at 0 h, 24 h, 72 h, 120 h, 168 h, and 336 h. The results are shown in Figure 23.

It can be seen from the figure that in the early stage of the experiment, both self-healing coatings have relatively good anti-corrosion effects, and there is no obvious corrosion phenomenon at the scratches.

However, in the later stage of the experiment, a large number of black corrosion marks appear under the ordinary self-healing coating film. The acidic pH-responsive self-healing coating has slight corrosion, and no edge corrosion or sub-film corrosion occurs during the entire experiment period. The reason is that the microcapsules in the ordinary self-healing coating only have a single wall material and are more likely to be lost during the coating preparation process. Therefore, the repair effect at scratches is also lower than that of the acidic pH-responsive self-healing coating. In addition, the stimulus responsiveness of the acidic pH-responsive self-healing coating enables the microcapsules in non-scratch areas to release the core material in time, inhibiting the occurrence of corrosion under the film.

The experimental results show that the acidic pH-responsive self-healing coating successfully improves the anti-corrosion ability of the coating by adding microcapsules with dual triggering modes of pH stimulus response and mechanical stimulus response.

#### 3.4.2. Research on Anti-Corrosion Properties of Coatings Based on Electrochemical Experiments

**Figure 24:**
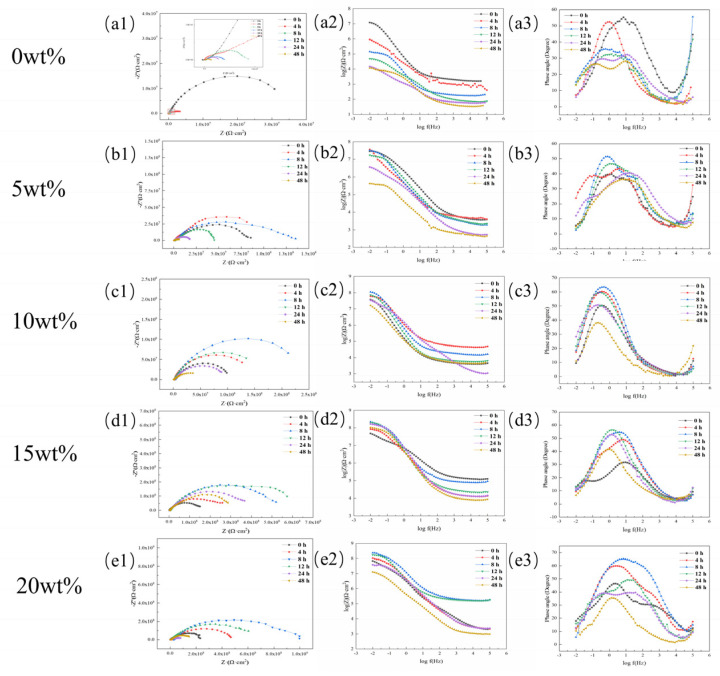
Nyquist plots and Bode plots of acidic pH−responsive self-healing coatings with different amounts of microcapsules added at different times: (**a**) 0 wt%; (**b**) 5 wt%; (**c**) 10 wt%; (**d**) 15 wt%; (**e**) 20 wt%.

To quantitatively study the anti-corrosion performance of the acidic pH-responsive self-healing coating, this experiment tested the change in the impedance spectrum of the microcapsule coatings containing 0 wt%, 5 wt%, 10 wt%, 15 wt%, and 20 wt% with soaking time. The test method is shown in Section 2.4.3. The results are shown in Figure 24.

EIS measurements can effectively monitor the self-healing process [35,36]. The low-frequency impedance value is the main indicator reflecting the protective performance of the coating. Generally speaking [37,38], when the low-frequency impedance value of the carbon steel/organic coating system is lower than 1 × 10^6^ Ω·cm^2^, the coating can be considered to have basically failed.

**Figure 25:**
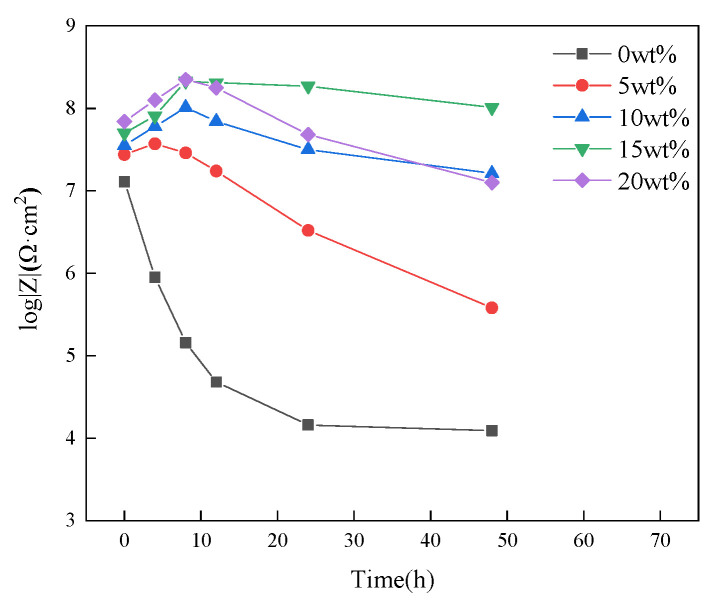
Low-frequency impedance values of acidic pH-responsive self-healing coatings at different times with different amounts of microcapsules added.

To visually display the change in the coating impedance value with soaking time, the low-frequency impedance value is drawn as a line graph, as shown in Figure 25. Comparative analysis shows that in the early stage of coating soaking, the amount of microcapsules added has a certain positive correlation with the low-frequency impedance value of the coating, which shows that the addition of microcapsules is beneficial for enhancing the corrosion shielding performance of the coating.

On the whole, the pure epoxy coating has the most obvious deterioration. During the entire experimental period, the impedance value and capacitive arc radius are the lowest values in the same period. This is because the epoxy coating cannot self-repair. After scratches, the corrosive medium can easily penetrate the coating through the damaged area, reducing the corrosion shielding ability of the coating.

**Figure 26:**
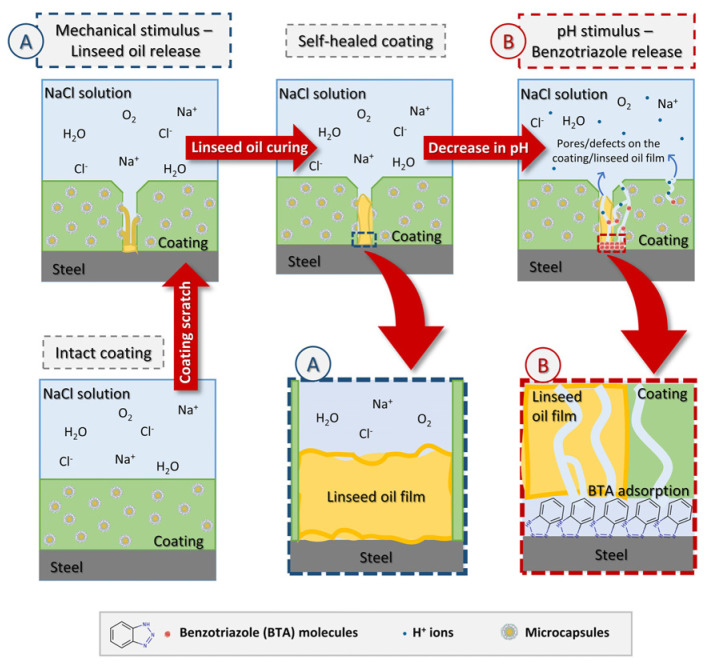
A schematic representation of the active corrosion protection mechanism provided by the coating loaded with microcapsules [39].

After adding microcapsules, the capacitive arc radius and low-frequency impedance value of the coating showed a trend of first increasing and then decreasing with time. This shows that the addition of microcapsules gives the coating the ability to self-heal. The self-healing mechanism of the coating in the sodium chloride etching medium can be explained in Figure 26; when the coating film is damaged by a mechanical stimulus, like a scratch, the microcapsules are ruptured, and the encapsulated linseed oil is released automatically. After linseed oil release, the oil film dries through a reaction with the dissolved O_2_ and forms a protective barrier film that diminishes the corrosion rate (acting as a healing agent) [39].

As the microcapsule content further increases, the coating has better self-healing capabilities. At 8 h, the coatings containing 15 wt% and 20 wt% microcapsules had low-frequency impedance values of 2.14 × 10^8^ Ω·cm^2^ and 2.24 × 10^8^ Ω·cm^2^, respectively (Figure 24d,e). During the entire soaking cycle, the coating with a microcapsule content of 15 wt% always maintained a high barrier capacity, and the low-frequency impedance value was still 1.02 × 10^8^ Ω·cm^2^ after 48 h.

However, the anti-corrosion performance of the coating with 20 wt% microcapsule content declined significantly after the soaking time continued to increase, reaching only 1.26 × 10^7^ Ω·cm^2^ at 48 h. It is an order of magnitude smaller than the 15 wt% coating with the same soaking time. The reason is that after a large number of microcapsules contained in the 20 wt% coating are released from the core material, the remaining empty shells form corrosion paths in the coating, causing the coating’s impedance value to drop rapidly.

Combining the results of the previous salt spray experiments and electrochemical tests, it can be found that the coating with 15 wt% microcapsules has the best anti-corrosion effect.

**Figure 27:**
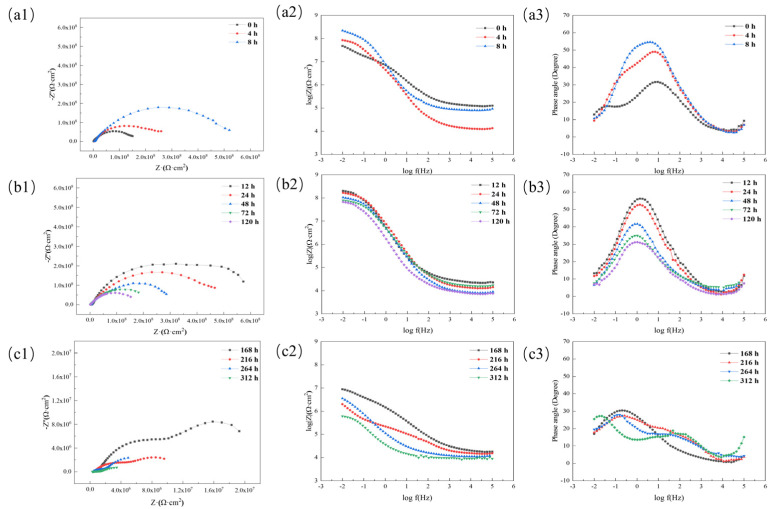
Nyquist diagram and Bode diagram of acidic pH−responsive self−healing coating at different times: (**a**) self−healing stage; (**b**) penetration stage; (**c**) failure stage.

To further study the changes in the anti-corrosion performance of the acidic pH-responsive self-healing coating over a long period, this experiment extended the immersion time to 312 h. The impedance spectrum of the 15 wt% coating was tested at 0 h, 4 h, 8 h, 12 h, 24 h, 48 h, 72 h, 120 h, 168 h, 216 h, 264 h, and 312 h. The results are shown in Figure 27.

**Figure 28:**
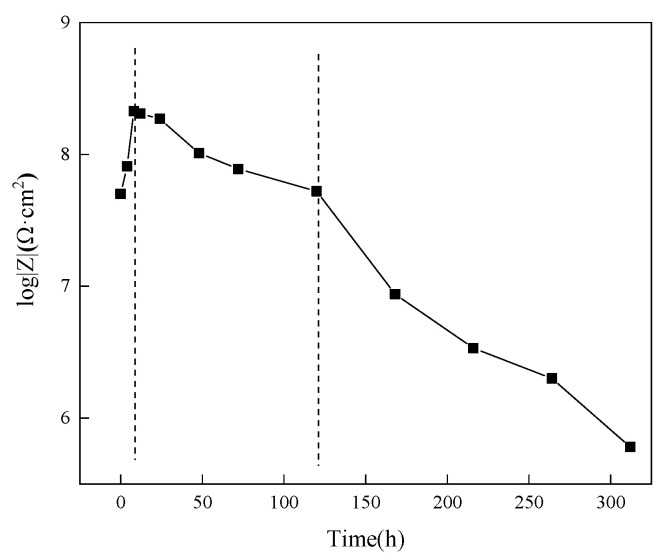
Low-frequency impedance value of acidic pH-responsive self-healing coating during long-term immersion experiment.

**Figure 29:**
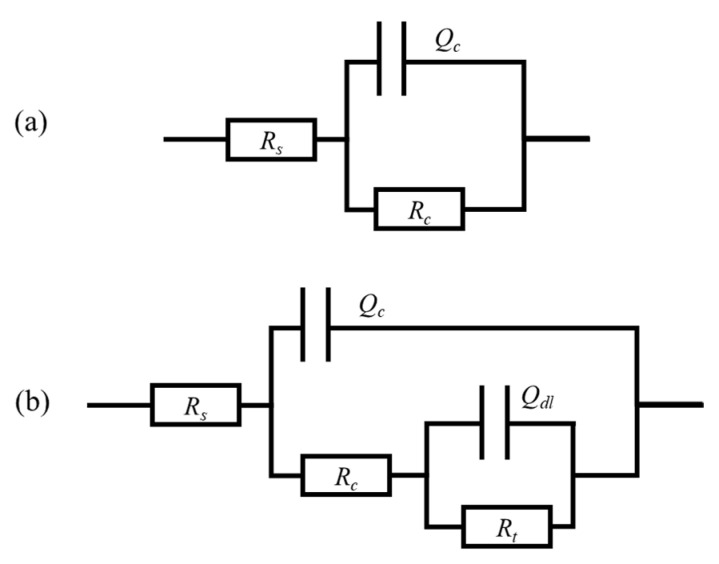
Equivalent circuit diagram during corrosion process of acidic pH-responsive self-healing coating: (**a**) self-repair stage, (**b**) failure stage.

According to the changes in the Nyquist diagram and Bode diagram during the experiment, the corrosion process of the coating is divided into three stages: self-repair stage (0~8 h), penetration stage (12~120 h), and failure stage (168~312 h). The change in the low-frequency impedance value of the coating with time is shown in Figure 28. To further study the corrosion mechanism of the coating, the equivalent circuit model in Figure 29 was used to fit the impedance spectrum of the coating.

In the self-healing stage, due to the oxidation of the linseed oil in the scratch and the protective film formed by the combination of benzotriazole with the metal substrate, the barrier performance of the coating steadily improved with time, reaching its maximum at 8 h. In the subsequent penetration stage, the repair process of the microcapsules was completed, the corrosive medium continued to penetrate the coating again, and the impedance and capacitive arc radii of the coating gradually decreased. The peak value of the phase angle decreased and moved to the low-frequency region. In both stages, the coating has good barrier properties, a higher resistance value, and a lower capacitance value, which is equivalent to pure resistance, so it can be simulated by the equivalent circuit in Figure 29a.

After 168 h, the coating began to show obvious double capacitive arc resistance, which indicates that the corrosive medium had completely penetrated. As the immersion time continued to increase, the peak phase angle of the coating decreased significantly, and double peaks appeared. The capacitive reactance arc moved toward the high-frequency end and decreased, and the low-frequency impedance value of the coating decreased rapidly. After immersion for 312 h, the resistance value of the coating dropped to 6.02 × 10^5^ Ω·cm^2^, which is already less than 10^6^ Ω·cm^2^. The impedance spectrum of the coating at this stage can be fitted by the circuit in Figure 29b.

**Figure 30:**
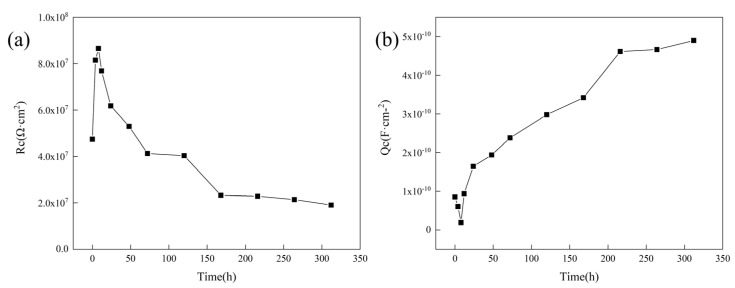
Coating resistance (**a**) and coating capacitance (**b**) during corrosion process of acidic pH−responsive self-healing coating.

In the equivalent circuit, *R*_s_ represents the resistance of the solution system, and *R*_C_ and *Q*_C_ represent the coating resistance and coating capacitance, corresponding to the time constant at the low-frequency end of the impedance spectrum. *R*_t_ and *Q*_dl_ represent the charge transfer resistance and double-layer capacitance of the electric double layer, corresponding to the time constant at the high-frequency end of the impedance spectrum. The electrochemical parameter fitting results obtained by fitting according to Figure 29 are shown in Table 1. To more intuitively display the penetration behavior of corrosive media in different corrosion stages, the changes in coating resistance and coating capacitance over time are plotted, as shown in Figure 30.

*R*_C_ and *Q*_C_ in the fitting parameters can be used to represent the anti-corrosion effect of the coating. *R*_C_ represents the resistance of the coating to electric current, which will decrease as the barrier ability of the coating decreases, while *Q*_C_ represents the ability of the coating to store charges, and its value will increase with the penetration of corrosive media. Through the study of these two parameters, it is possible to evaluate changes in the corrosion protection ability of the coating over time.

During the soaking cycle, *R*_C_ showed a trend of first increasing and then decreasing, while *Q*_C_ continued to increase after first decreasing. This is because in the early stage of immersion, the coating exhibits self-healing behavior, repairing scratches and minor defects and enhancing its barrier capability. After the self-healing process is completed, the corrosive medium penetrates the interior of the coating again, which has a large dielectric constant, causing *R*_C_ to decrease rapidly. As the immersion time further increases, corrosion reactions occur at the coating interface, and the holes and gaps in the coating are blocked by corrosion products. The *R*_C_ of the system decreases slowly and becomes stable at 168 h.

Combining the test results of *R*_C_ and *Q*_C_, it can be found that the anti-penetration ability of the coating first increased and then decreased during the immersion process, and the coating was completely penetrated at 168 h.

#### 3.4.3. Study of the Difference in Anti-Corrosion Performance of Self-Healing Coatings in Acid and Alkali Environments

**Figure 31:**
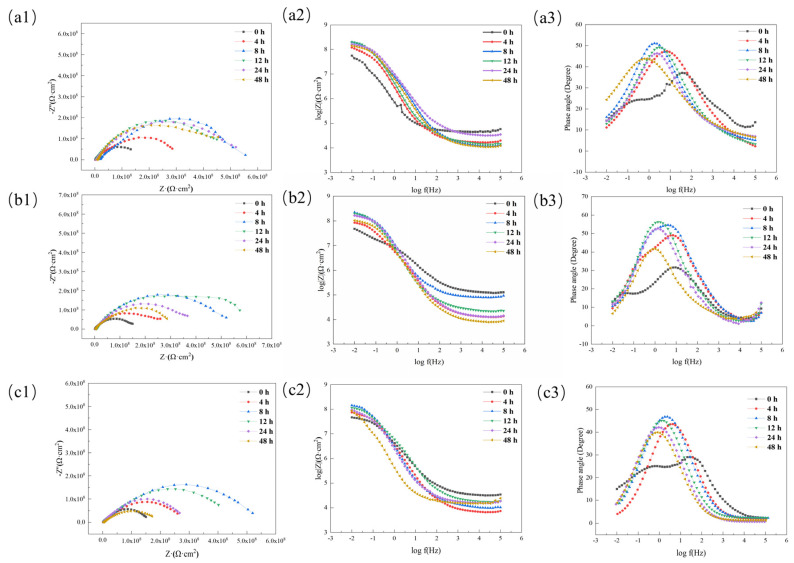
Nyquist plots and Bode plots of acidic pH-responsive self−healing coatings at different times under different pH environments: (**a**) pH = 5; (**b**) pH = 7; (**c**) pH = 9.

To further study the impact of the acidic pH stimulus response function on the anti-corrosion performance of the coating, changes in environmental pH at the cathode and anode reaction sites on the metal substrate in the coating were simulated by changing the pH value of the electrochemical test solution. The anti-corrosion performance changes of the acidic pH-responsive intelligent self-healing coating with scratches during immersion in solutions with different pH were tested, and the electrochemical results were compared with the electrochemical results of the ordinary self-healing coatings with scratches with the same microcapsule content. The results are shown in Figure 31.

It can be seen that after being immersed in solutions with different pH values, the impedance spectra of the coatings change in similar patterns, showing a trend of repairing first and then penetrating. However, there are differences in the repair speed and post-repair barrier capabilities of the coating under different pH values. Comparing the impedance value and capacitive arc change of the coating in three different environments, it can be found that when the environmental pH is 5, the impedance value of the coating is greater than that of the other two groups of coatings in the same period. The analytical reason is that in acidic media, the shell of the microcapsules in the coating is protonated and the internal core material is quickly released. The microcapsules that respond to mechanical stimulation and the microcapsules that respond to pH stimulation have a synergistic anti-corrosion effect, causing the impedance value of the coating to increase rapidly and then remain stable, still being 1.62 × 10^8^ Ω·cm^2^ after 48 h.

When the environmental pH increases, the anti-corrosion performance of the coating decreases in the early stage and declines after 48 h, but it is still greater than the failure value of 1 × 10^6^ Ω·cm^2^. The reason is that under neutral and alkaline conditions, only the core material of the microcapsules at the scratched area is released by mechanical stimulation, the overall release rate is low, and there are still many tiny defects in the coating.

In summary, the coating achieves basic self-healing and anti-corrosion effects by responding to mechanical stimuli when the environmental pH is alkaline and neutral. When the environmental pH changes to acidic, the coating has a better repair effect through a dual-trigger stimulus response mechanism.

## 4. Conclusions

The preparation process of acidic pH-responsive microcapsules was prepared and optimized through layer-by-layer assembly of polyelectrolyte materials. The prepared pH-responsive microcapsules have good morphology, an average particle size of approximately 203 μm, a drug loading rate of 59.74%, and an encapsulation rate of 63.99%. The sustained release curve test results of the microcapsules show that the optimized microcapsules have good pH sensitivity, can remain stable in neutral and alkaline environments, and rapidly release the core material in acidic environments. The thermogravimetric analysis results of the microcapsules show that the pH-responsive microcapsules have good thermal stability and can meet the basic needs of anti-corrosion coatings. The self-healing effect of the acidic pH-responsive intelligent self-healing coating is proportional to the amount of microcapsules added. When the addition amount exceeds 15 wt%, the repair efficiency of the coating is good. Based on the results of the scratch test, salt spray test, and impedance spectroscopy test, it can be obtained that the optimal additional amount of microcapsules is 15 wt%. The coating has an obvious stimulus response to acidic pH and can inhibit corrosion at non-scratch locations. Compared with ordinary self-healing coatings and epoxy coatings, acidic pH-responsive intelligent self-healing coatings have stronger protective capabilities. The acid–base immersion test results show that the acidic pH-responsive intelligent self-healing coating still has a good anti-corrosion effect after the environmental pH value changes. When the environmental pH value changes to acidic, the anti-corrosion ability of the coating is better.

## Figures and Tables

**Table 1:** Equivalent circuit fitting values of acidic pH-responsive self-healing coatings.

Stage	Time (h)	*R*_s_ (Ω·cm^2^)	*Q*_c_ (F·cm^−2^)	*R*_c_ (Ω·cm^2^)	*Q*_dl_ (F·cm^−2^)	*R*_t_ (Ω·cm^2^)
Self-healing	0	4493	8.5381 × 10^−11^	4.74 × 10^7^	-	-
	4	4672	6.0461 × 10^−11^	8.15 × 10^7^	-	-
	8	6157	1.9 × 10^−11^	8.66 × 10^7^	-	-
Infiltration	12	6559	9.3759 × 10^−10^	7.69 × 10^7^	-	-
	24	5793	1.6446 × 10^−10^	6.18 × 10^7^	-	-
	48	6534	1.9379 × 10^−10^	5.30 × 10^7^	-	-
	72	3074	2.3812 × 10^−10^	4.12 × 10^7^	-	-
	120	3127	2.9789 × 10^−10^	4.04 × 10^7^	-	-
Failure	168	2667	3.420 × 10^−10^	2.32 × 10^7^	1.26 × 10^−10^	4.07 × 10^7^
	216	5206	4.6131 × 10^−10^	2.28 × 10^7^	1.36 × 10^−10^	5.28 × 10^6^
	264	2771	4.6644 × 10^−10^	2.14 × 10^7^	3.64 × 10^−10^	7.87 × 10^6^
	312	6636	4.899 × 10^−10^	7.04 × 10^6^	1.32 × 10^−10^	2.05 × 10^6^

## Data Availability

The original contributions presented in the study are included in the article, further inquiries can be directed to the corresponding author/s.
